# Voretigene Neparvovec Gene Therapy in Clinical Practice: Treatment of the First Two Italian Pediatric Patients

**DOI:** 10.1167/tvst.10.10.11

**Published:** 2021-09-23

**Authors:** Francesco Testa, Paolo Melillo, Michele Della Corte, Valentina Di Iorio, Raffaella Brunetti-Pierri, Amelia Citro, Maurizio Ferrara, Marianthi Karali, Rosa Annibale, Sandro Banfi, Settimio Rossi, Francesca Simonelli

**Affiliations:** 1 Eye Clinic, Multidisciplinary Department of Medical, Surgical and Dental Sciences, University of Campania Luigi Vanvitelli, Naples, Italy; 2 Anesthesiology Unit, Azienda Ospedaliera Universitaria, University of Campania Luigi Vanvitelli, Naples, Italy; 3 Telethon Institute of Genetics and Medicine, Pozzuoli, Italy; 4 Pharmacy Unit, Azienda Ospedaliera Universitaria, University of Campania Luigi Vanvitelli, Naples, Italy; 5 Medical Genetics, Department of Precision Medicine, University of Campania Luigi Vanvitelli, Naples, Italy

**Keywords:** *RPE65*-related inherited retinal dystrophy, voretigene neparvovec, gene therapy, visual acuity, semiautomatic kinetic visual field, full-field stimulus threshold test, microperimetry, chromatic pupillometry

## Abstract

**Purpose:**

To present visual outcomes of the first two Italian patients with *RPE65*-related inherited retinal dystrophy (*RPE65*-IRD) treated with voretigene neparvovec (VN).

**Methods:**

Two pediatric patients with *RPE65*-IRD were treated with VN in both eyes. Patients were evaluated by best-corrected visual acuity (BCVA), full-field stimulus threshold (FST) test, semiautomated kinetic visual field (SKVF), microperimetry, and chromatic pupillometry over 6 months.

**Results:**

No complications occurred in the first patient, whereas in the second a subretinal hemorrhage was observed in the first treated eye, and excessive resistance to drug injection occurred during treatment of the second eye. BCVA improved by at least one Early Treatment Diabetic Retinopathy Study line in all treated eyes. The FST test and SKVF showed clinically significant improvements in all eyes (i.e., change of light sensitivity > 10 decibels; area enlargement of at least 20%). Moreover, microperimetry showed better fixation stability. Finally, chromatic pupillometry showed increases in pupillary constriction that ranged from 10% to 20%. All visual changes remained stable during follow-up.

**Conclusions:**

The first VN treatments in two pediatric Italian patients in clinical practice showed significant improvements in visual outcomes, even in the case of surgical complications, which spontaneously recovered without sequelae.

**Translational Relevance:**

These findings with VN in patients with *RPE65*-IRD confirm the results of clinical trials.

## Introduction

Biallelic mutations in *RPE65* are associated with severe, rod-mediated inherited retinal dystrophy, which presents as night blindness in early childhood that eventually progresses to complete blindness.^1^^–^^3^ In 2008, gene therapy with unilateral injection of the recombinant adenoviral vector voretigene neparvovec (VN) in three patients with *RPE65*-related inherited retinal dystrophy (*RPE65*-IRD) was performed, and improvements in visual function were noted.^4^ Following phase 1 and 2 trials, an open-label, randomized, controlled phase 3 trial was carried out in 31 patients with *RPE65*-IRD.^5^ After 1 year, improvements were seen in light sensitivity and visual fields, as well as navigational ability under dim lighting conditions. Based on these results, in 2017 VN was approved by the US Food and Drug Administration for treatment of patients with *RPE65*-IRD and in 2018 by the European Medicines Agency for the same indication.

To date, there is little experience in clinical practice with VN in treatment of *RPE65*-IRD, and outcomes and surgical techniques in only small numbers of patients have been reported.^6^^–^^8^ Herein, we present visual outcomes of the first two Italian patients with *RPE65*-IRD treated with VN.

## Materials and Methods

The study adhered to the tenets of the Declaration of Helsinki and was approved by the Ethics Committee of the University of Campania Luigi Vanvitelli. Written assent from both patients and informed consent from parents were obtained. Two patients with confirmed *RPE65*-IRD were treated with VN from December 2019 to January 2020. The first patient (patient 1) is a 9-year-old male with biallelic mutations in *RPE65*: p.His313Arg/p.Asp482Val. The second patient (patient 2) is an 8-year-old female with biallelic mutations in *RPE65*: p.His313Arg/p.Tyr368His. Treatment expenses were reimbursed by a national fund (Fondo AIFA 5%) that supplies medicines awaiting market entry to patients with rare diseases.

Both patients were evaluated before and after surgery at 1, 2, 6, 15 (only first treated eye), 30 (only second treated eye), 45 (only first treated eye), 120, and 180 days. The day 30 follow-up visit of the second eye of patient 2 was postponed to day 60 because of restrictions related to the COVID-19 pandemic.

The effects of treatment on visual function were assessed using best-corrected visual acuity (BCVA), full-field stimulus threshold (FST) test, semiautomated kinetic visual field (SKVF), fixation stability evaluation by microperimetry, and chromatic pupillometry performed before and at the post-treatment time points starting from day 30. Moreover, BCVA, SKVF, and pupillometry were also performed on day 15 after treatment of the first eye. Moreover, changes in retinal morphology were evaluated by indirect ophthalmoscopy, color fundus imaging, and optical coherence tomography (OCT) performed at each time point. BCVA was measured using a standard protocol involving Early Treatment Diabetic Retinopathy Study (ETDRS) charts; letter scores were converted to the log of the minimum angle of resolution (logMAR).

SKVF testing was performed using an Octopus 900 Pro Perimeter instrument with Eye Suite i4.000 software (Haag-Streit International, Koeniz, Switzerland). Background illumination of the bowl was 31.4 apostilbs. Data were collected using stimulus test sizes V4e, III4e, and I4e, and total seeing area was calculated for each isopter (seeing area minus defined scotoma). Test vectors were presented approximately every 15° at an angular velocity of 4°/s and originating approximately 10° outside the age-correlated normal isopter. Scotomas were mapped using an angular velocity of 2°/s, originating from the assumed center using at least 12 vectors. Blind-spot mapping was done with the smallest test size target. A change of 20% in seeing area using stimulus sizes III4e and V4e was considered clinically meaningful, as previously suggested in patients with inherited retinal dystrophies.^9^

The FST test, conducted using the Espion E3 ColorDome (Diagnosys LLC, Lowell, MA),^10^ measures luminance thresholds using full-field stimuli generated by narrow-band light-emitting diodes (LEDs). Stimuli below 0.01 cd·s/m^2^ (“dim” LEDs) had peak wavelengths of 468 nm (blue) and 632 nm (red); stimuli above 0.01 cd·s/m^2^ (“bright” LEDs) were 444 nm (blue) and 632 nm (red). A button box was used by the subject to indicate if a brief, full-field stimulus was perceived. To perform the test, pupils were dilated, and the FST test was performed in the dark after 40 minutes of dark adaptation, first testing with a blue stimulus followed by 6500K white and red stimuli. A 10-decibel (dB) change was considered clinically meaningful.^10^^,^^11^

Fixation stability was evaluated with an automatic fundus-related perimeter (MP1 Microperimeter; Nidek Technologies, Padova, Italy) using the following parameters: fixation target of 2° in diameter consisting of a red cross and a white, monochromatic background with luminance of 1.27 cd/m^2^. Fixation stability was assessed in terms of percentage of fixation points that fell within a 2° (FS2°) and 4° diameter (FS4°) circle, as well as bivariate contour ellipse area (BCEA), as previously described.^12^

The protocol for chromatic pupillometry was defined as reported^13^^–^^18^ using a binocular pupillometer (DP-2000; NeurOptics, Irvine, CA). Measurements started after 20 minutes of dark adaption. The protocol was based on 1-second stimuli presented to each eye at three intensities (0 log lux, 1 log lux, and 2 log lux) and colors (red, λ = 622 nm; blue, λ = 462 nm; and white) and repeated twice. Pupillary responses were automatically analyzed by the pupillometer and manually reviewed to extract the maximum pupillary constriction.

### Procedures

Patients received 1 mg/kg/d (up to 40 mg/d) of prednisone orally for 7 days, beginning 3 days before their first injection. Prednisone was tapered (0.5 mg/kg/d, up to 20 mg/d) for the following 7 days or until 3 days before injection of the second eye, when the steroid regimen was repeated. The surgical technique was OCT-guided (Proveo 8 ophthalmic microscope; Leica Microsystems, Wetzlar, Germany) 25-gauge vitrectomy (CONSTELLATION Vision System; Alcon, Geneva, Switzerland). Sclerotomies were performed at 3.5 mm from the limbus. Following standard core vitrectomy, posterior vitreous detachment was induced with preservative-free triamcinolone acetonide. Complete vitrectomy was performed with a 360° depressed shave, and the surgeon checked that no peripheral ruptures or detachments, which could compromise surgical outcomes, had occurred. Next, single subretinal injection of VN was performed along the upper vascular arcades, avoiding vascular structures and areas of atrophy, at least 2 mm away from the center of the foveal, using a 25/38-gauge needle (PolyTip Cannula 25g/38g; MedOne Surgical, Sarasota, FL). As recommend in the Summary of Product Characteristics, patients were expected to receive a single dose of 1.5 × 10^11^ vector genomes VN in each eye, and each dose was planned to be delivered into the subretinal space in a total volume of 0.3 mL. The procedure was carried out with the support of OCT to more accurately perceive detachment of the posterior vitreous before carrying out the procedure, as well as the injection site and presence of the subretinal bubble determined by injection of the vector. After satisfactory delivery of the vector, a fluid–air exchange was performed to remove any drug from the posterior segment to minimize postoperative inflammation and to improve dissemination of the drug. Finally, sclerotomies were sutured with vicryl 7/0, and a subconjunctival injection of betamethasone and gentamicin was given.

## Results

### Patient 1

In this patient, the worst eye (left) was treated first, and the right eye was treated 14 days later. There were no complications during treatment of either eye, and no signs of intraocular inflammation were observed over follow-up. At baseline, BCVA was 20/100 in the right eye and 20/125 in the left eye, with a correction of +3 diopters (D) in both eyes. At 15 days from treatment in the first treated eye (left), BCVA in the left eye was 20/80 with improvement of two ETRDS lines. Similarly, at the first evaluation (30 days) after treatment of the second eye (right), BCVA was 20/80 in both eyes, with an improvement of one ETDRS line in the right eye. BCVA remained stable over 6 months.

**Figure 1.**
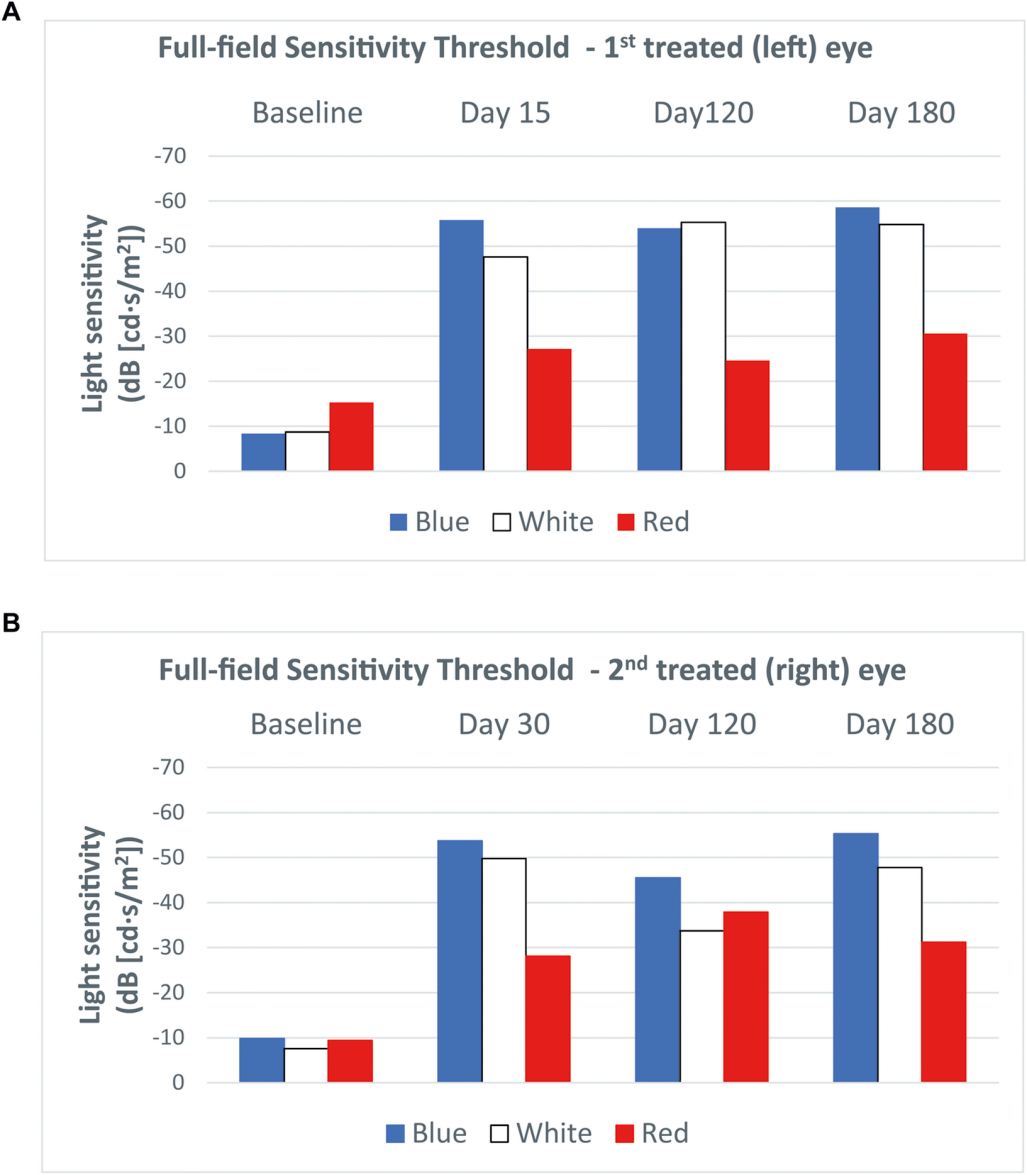
FST in patient 1 in the first (left) eye treated (a) and the second (right) eye treated (b). Clinically significant improvements (i.e., >10 dB) of the light sensitivity threshold in response to all three stimulus colors were observed in both eyes at the earliest post-treatment time point.

The FST test at 15 days after treatment of the left eye showed clinically significant improvements of light threshold of 47.4 dB, 38.9 dB, and 11.9 dB for blue, white, and red light stimuli, respectively. In the right eye, at the earliest time point (30 days) after treatment, the light threshold in response to blue, white, and red light stimuli improved by 43.9 dB, 42.2 dB, and 18.7 dB, respectively. These improvements were stable at 6 months (Fig. 1).

**Figure 2.**
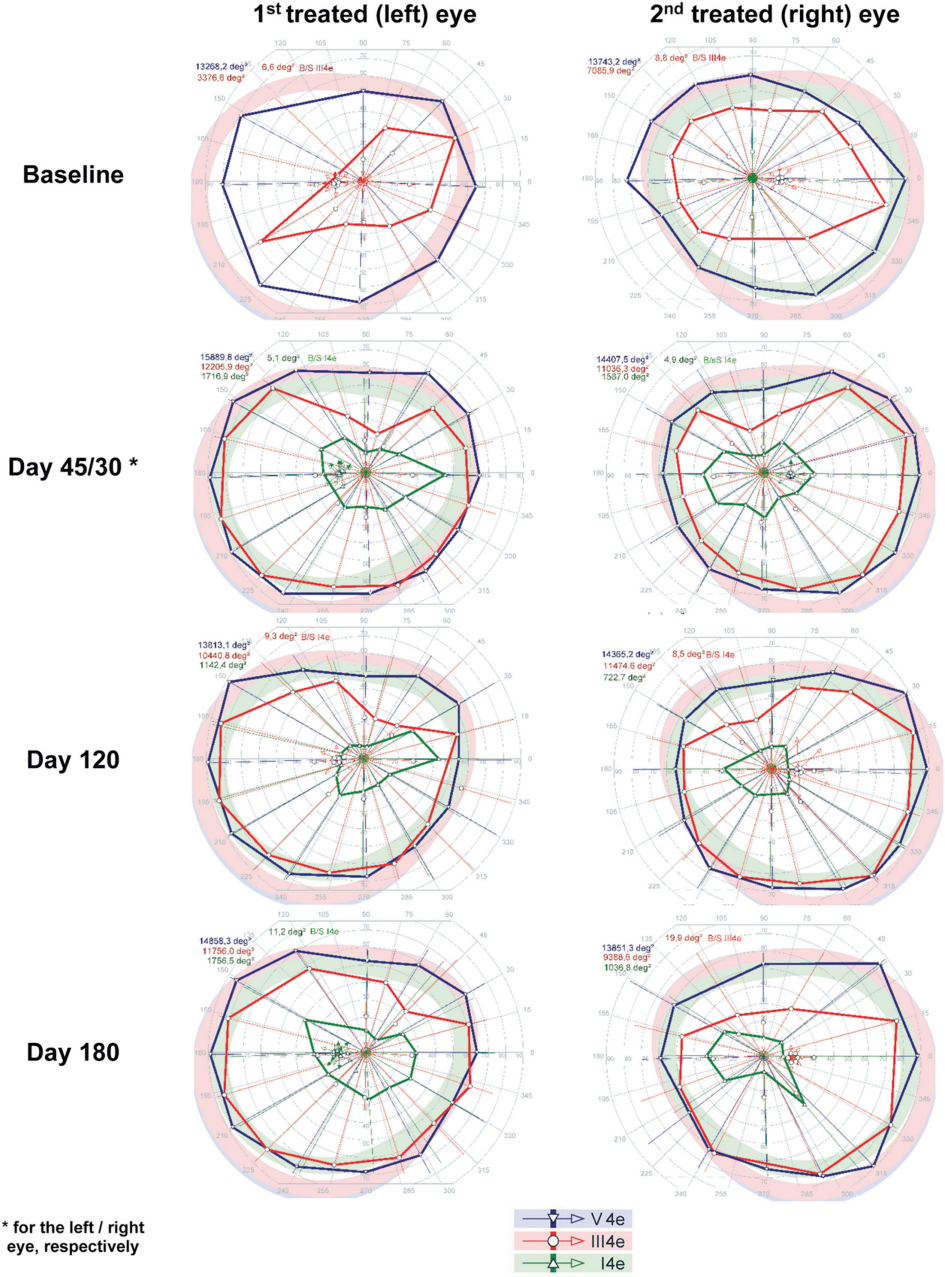
Visual field before and after treatment in patient 1 showing clinically significant enlargement of III4e isopters in both eyes and responses to I4e stimuli after treatment.

SKVF before treatment showed a markedly reduced area in response to III4e stimuli (right eye, 11,036.3°^2^; left eye, 3376.6°^2^) and a slightly reduced area in response to V4e stimuli compared with normal (right eye, 14,407.5°^2^; left eye, 14,365.2°^2^), although the patient did not see the smallest stimulus size analyzed (i.e., I4e) in either eye. Figure 2 shows the SKVF before and after treatment. At day 45, a dramatic increase of SKVF area in response to III4e stimuli higher than 200% was observed in the left eye that remained stable throughout follow-up, whereas the SKVF area in response to V4e stimuli increased, even if the improvement was less marked (19.8% at day 45 and 12.0% at day 180). After treatment, the patient was able to see the smallest stimulus size analyzed (i.e., I4e). Regarding the right eye, at the earliest time point (day 30), there was an increase in SKVF in response to III4e stimuli higher than 50%, which slightly decreased at day 180 (32.5%). In response to the highest stimulus size (i.e., V4e), smaller changes (lower than 5%) were observed over 6 months, whereas after treatment the patient was able to see the smallest stimulus size analyzed (i.e., I4e) even with the right eye.

**Figure 3.**
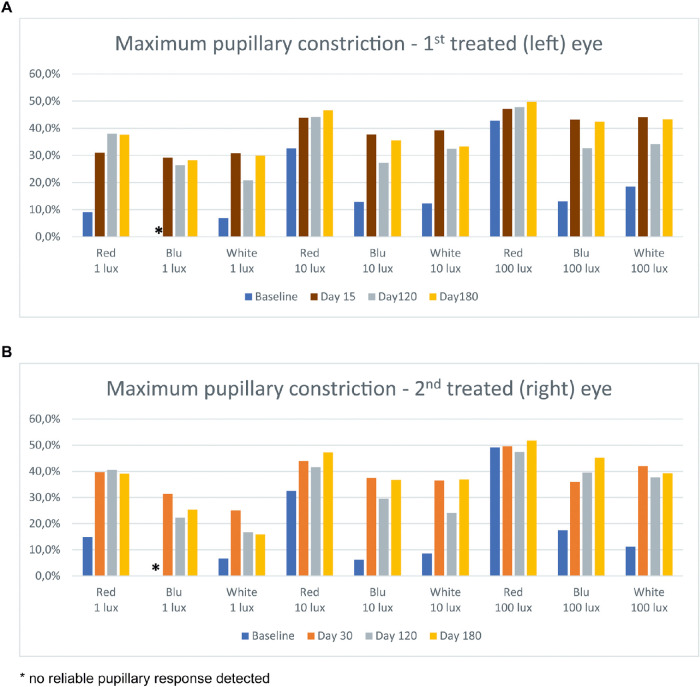
Results of chromatic pupillometry in patient 1 in the first (left) eye treated (a) and the second (right) eye treated (b) showing an increase of maximum pupillary constriction (about 10%–20%) in response to all light stimuli (except high-intensity red) since the earliest post-treatment time points.

At the earliest post-treatment time points (15 days and 30 days for the left and right eye, respectively), chromatic pupillometry showed an increase of maximum pupillary constriction (about 20%) in response to blue and white light stimuli at all tested lux levels and to low-intensity red stimulus, whereas less relevant changes (about 10%) were observed in response to middle-intensity red light stimulus (Fig. 3). These improvements were maintained at 6 months, with a maximum pupillary constriction higher than baseline of about 10%.

At baseline, motility evaluation highlighted anomalous head position and alternating esotropia, whereas after treatment (day 120) improvement of head position was observed together with alternating esophoria. Regarding fixation stability, at baseline the patient had unstable fixation in the left eye (FS2, 15%; FS4, 36%; BCEA area 68.2%, 9.43°^2^) and stable fixation in the right eye (FS2, 94%; FS4, 98%; BCEA area 68.2%, 0.78°^2^). At 6 months, stable fixation was present in both eyes with dramatic improvements of fixation stability parameters in the left eye (FS2, 97%; FS4, 100%; BCEA area 68.2%, 0.86°^2^) that were comparable in the right eye (FS2, 94%; FS4, 100%; BCEA area 68.2%, 0.99°^2^). A noteworthy observation in patient 1 is that, after treatment of the left eye, for the first time in his life he was able to play soccer with his brother outdoors on winter evenings with poor light illumination.

### Patient 2

**Figure 4.**
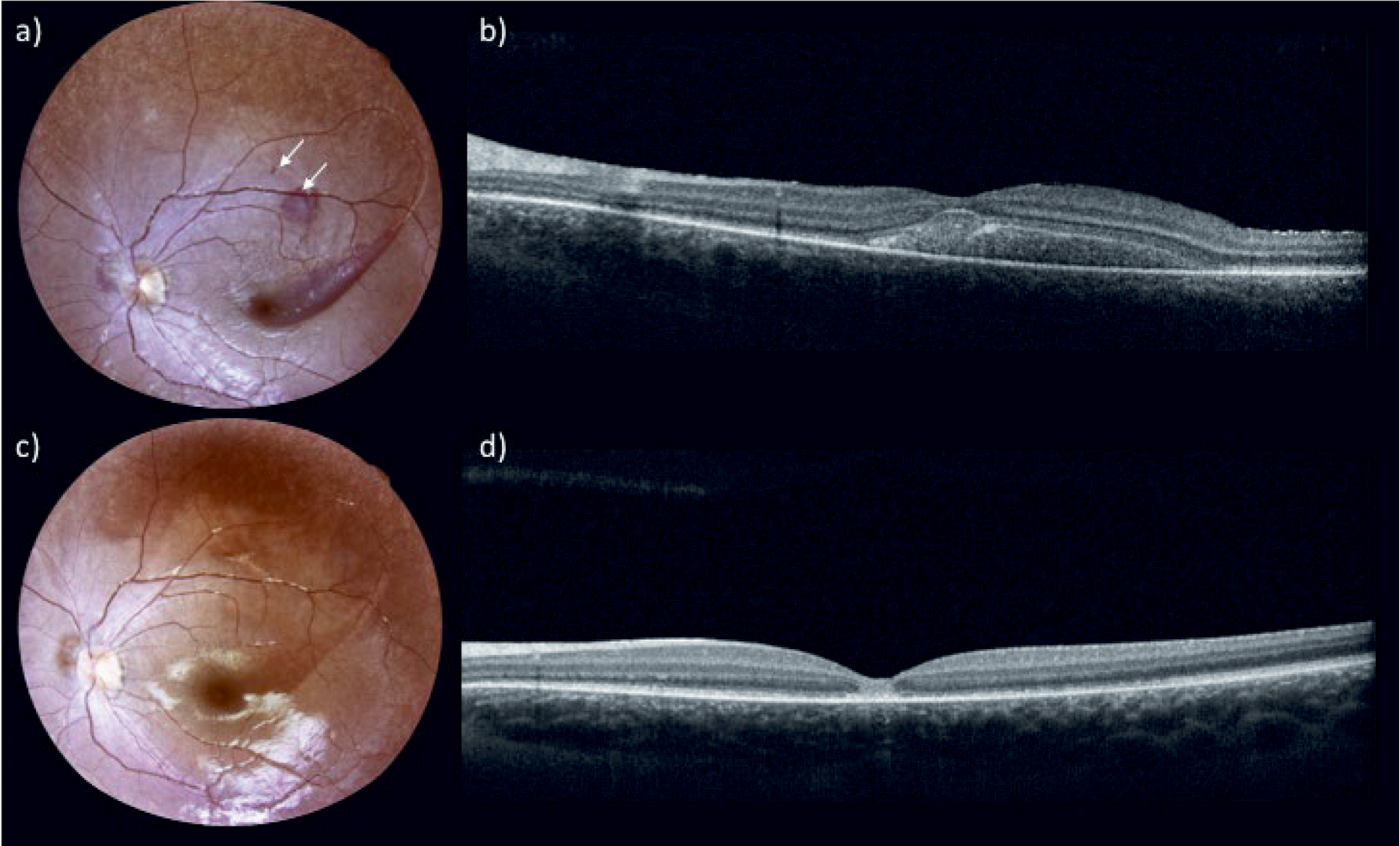
Fundus examination and OCT scan in the first treated eye of patient 2 at 1 day (a, b) and at 45 days (c, d) after treatment. One day after treatment, fundus examination (a) revealed a subretinal hemorrhage in the macular area, and the *arrows* indicate the sites of the two retinotomies. The OCT scan (b) revealed the persistence of a shallow retinal detachment with blood collection. Forty-five days after treatment, fundus examination (c) and OCT scan (d) confirmed resolution of the complication.

In this patient, the worst (left) eye was treated first, and the right eye was treated 45 days later. In both procedures, two injections were given; therefore, two blebs were made due to an initial reflux of the drug, excessive resistance to the introduction of the drug, and formation of a first bleb of unsuitable size. In particular, during injection in the left eye, subretinal hemorrhage with shallow retinal detachment was observed. For this reason, the injection was interrupted to perform a second retinotomy in another retinal site, nasally to the first, and the remaining dose was injected. One day after treatment, fundus examination and OCT scan revealed the persistence of a shallow retinal detachment with blood collection (up to day 15) (Fig. 4). One month after treatment, fundus examination and OCT scans confirmed resolution of the complication. During injection in the right eye, an initial reflux of the drug, excessive resistance to the introduction of the drug, and formation of a first bleb of unsuitable size required a second retinotomy (nasally to the first), which showed similar difficulties with a small retinal bleb (less than two papillary disks). No signs of intraocular inflammation were observed over follow-up in either eye.

At baseline, the patient showed a BCVA of 20/125 in the right eye and 20/320 in the left eye, with correction of +3 D in both eyes. In the left eye, BCVA remained stable up to day 15, and after resolution of subretinal hemorrhage it improved by three ETDRS lines at day 45 with further improvement of two additional ETDRS lines over the 6-month follow-up. In the right eye, less improvement (one ETDRS line) was observed starting at day 60. Following this improvement, the BCVA of 20/100 remained stable for up to 6 months.

**Figure 5.**
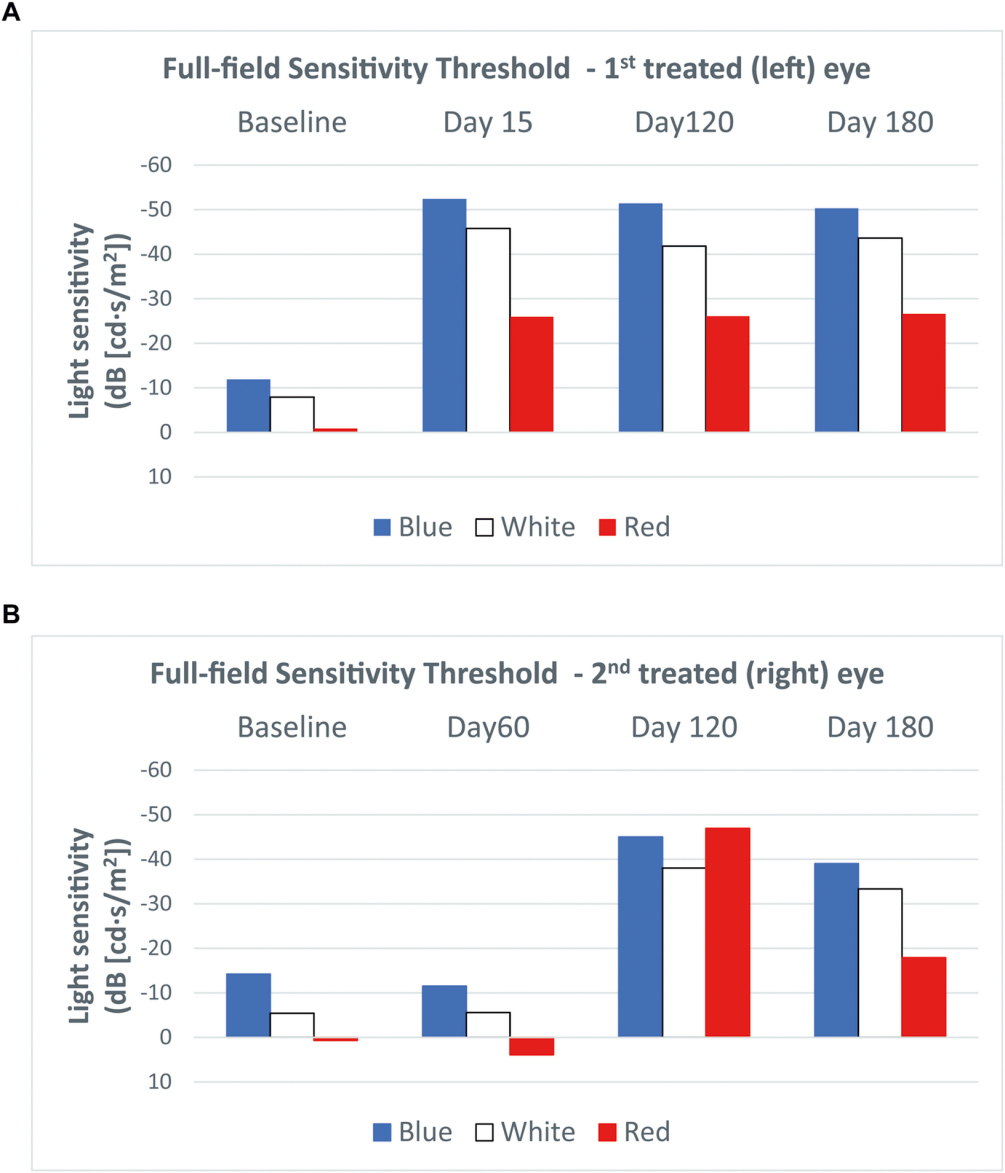
The FST in patient 2 in the first (left) eye treated (a) and the second (right) eye treated (b). Clinically significant improvements (i.e., >10 dB) of light sensitivity threshold in response to all three stimulus colors were observed in the first treated eye after day 15 and in the second treated eye after day 120.

The FST test performed 15 days after treatment of the left eye showed clinically significant improvements of light threshold of 40.5 dB, 37.9 dB, and 25.1 dB for blue, white, and red light stimuli, respectively, that remained stable over 6 months. In the right eye, improvements were observed starting from day 120 with clinically significant changes of light threshold of 30.8 dB, 32.6 dB, and 47.6 dB for blue, white, and red light stimuli, respectively. The last examination showed a non-clinically significant (i.e., <10 dB) reduction of improvements in blue and white light stimuli and marked reduction of the threshold to red light compared with day 120, even if the improvements versus baseline remained clinically significant (Fig. 5).

**Figure 6.**
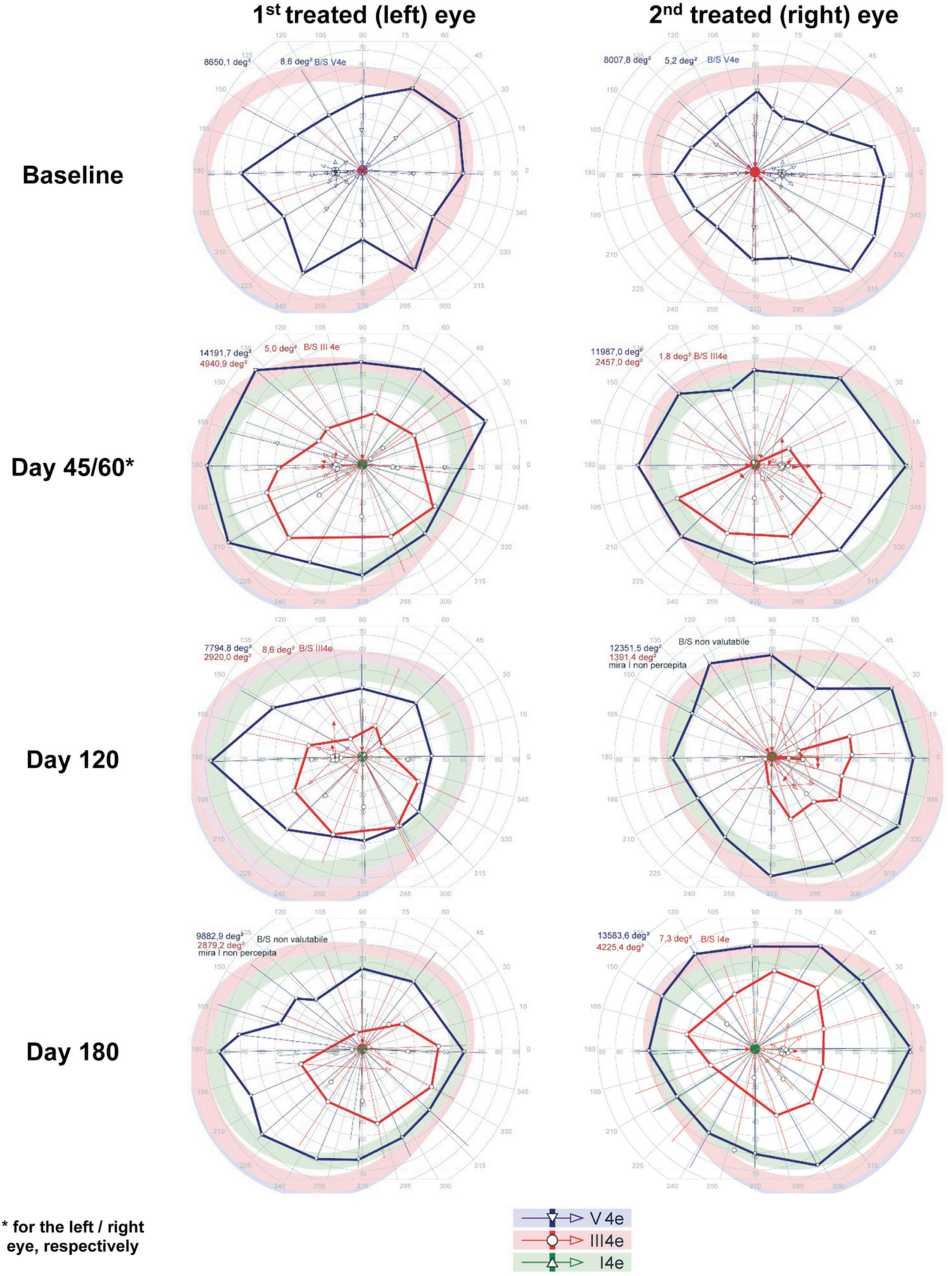
The visual field before and after treatment in patient 2 showing clinically significant enlargement of V4e isopters in both eyes and responses to III4e stimuli after treatment.

SKVF performed before treatment showed a markedly reduced area in response to V4e stimuli (right eye, 8007.8°^2^; left eye, 8650.1°^2^), whereas the patient did not see III4e or I4e stimuli. Figure 6 shows the SKVF before and after treatments. At day 45, a significant increase of VF area in response to V4e stimuli greater than 50% was observed in the left eye, even if at the last time point the area was only slightly (14.3%) larger than baseline. Furthermore, after treatment the patient was able to see stimulus size III4e, which remained stable during follow-up. Regarding the right eye, at the earliest time point (day 60), there was an increase of SKVF area in response to V4e stimuli of 49.7%, which increased to 69.6% by day 180. Finally, the patient was able to see stimulus size III4e, and the relative area increased at 6 months.

**Figure 7.**
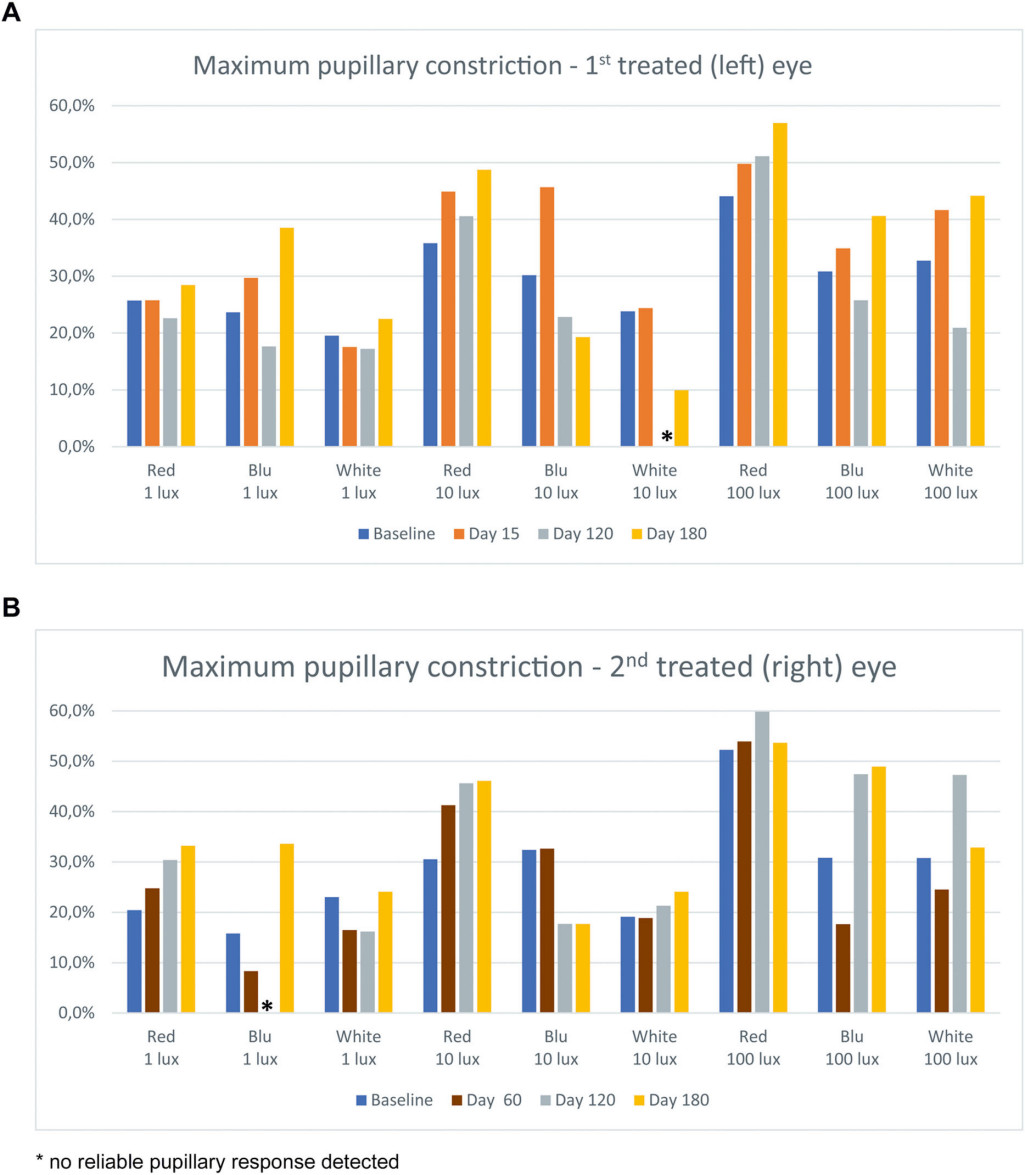
Results of chromatic pupillometry in patient 2 in the first (left) eye treated (a) and the second (right) eye treated (b), showing an increase of maximum pupillary constriction (10%–20%) in response to blue low-intensity, red middle-intensity, and all high-intensity stimuli since the earliest post-treatment time points.

At 6 months, compared with baseline, chromatic pupillometry showed an increase of maximum pupillary constriction (10%–15%) in response to blue low-intensity, red middle-intensity, and all high-intensity stimuli in the left eye (Fig. 7). In the right eye, an increase of maximum pupillary constriction (10%–20%) was observed in response to red and blue low-intensity, red middle-intensity, and blue high-intensity stimuli.

At baseline, motility evaluation highlighted nystagmus, anomalous head position, upshot, and alternating esotropia, consistent with an unstable fixation in both eyes shown by fixation testing performed by microperimetry. After treatment, fixation evaluation highlighted improvement of fixation stability in the left eye, changing from unstable (FS2, 1%; FS4, 12%; BCEA area 68.2%, 99.0°^2^) to relatively stable (FS2, 52%; FS4, 86%; BCEA area 68.2%, 5.6°^2^). Slight improvements in fixation parameters were also observed in the right eye (FS2, 2%; FS4, 4%; BCEA area 68.2%, 224.6°^2^ at baseline vs. FS2, 1%; FS4, 20%; BCEA area 68.2%, 45.4°^2^ at 6 months), although the fixation was unstable. A noteworthy observation in patient 2 is that, after treatment of the first eye, as reported by her relatives, for the first time in her life she was able to walk alone outdoors in her town in the evening and at nighttime.

## Discussion

Herein, we present outcomes at 6 months of the first two Italian pediatric patients with *RPE65*-IRD who underwent treatment with VN. The surgical procedures were performed without any intraoperative complication in patient 1, whereas subretinal injections in patient 2 required a second bleb to achieve satisfactory delivery of VN. In particular, the subretinal hemorrhage, which occurred in the first treated eye, and the excessive resistance to the introduction of the drug encountered in both procedures may be related to excessive deep penetration of the needle tip, despite the use of intraoperative OCT. Actually, as described in previous studies,^19^^,^^20^ a technical issue of intraoperative OCT has been the cannula shadow; the cannula obscured the needle tip penetrating the retina, and this made it difficult to evaluate the depth of needle penetration. Moreover, as suggested by Vasconcelos et al.,^20^ who reported a large case series of OCT-guided subretinal gene therapy surgery, the degree of retinal atrophy in the area of injection may also interfere with those complications.

During follow-up, there were no safety issues, and both patients showed improvements in both eyes, albeit to different extents. Improvements, ranging from one to five ETDRS lines, were seen in BCVA that were stable over 6 months of follow-up. Our results are largely consistent with the phase 3 trial with VN in which a treatment difference of 0.16 logMAR was reported, corresponding to roughly 1.5 ETDRS lines.^11^

We also observed significant benefit in the FST within 15 days in both eyes in patient 1 and in the first eye in patient 2, whereas in the second eye improvement was not seen until day 120. In all eyes, these changes were stable throughout 6 months. The magnitude of improvement in the FST was highly similar to that reported in an analysis of phase 1 and phase 3 trials.^21^ As noted, the improvement in the FST was most evident for blue light, which is expected given the enhancement of rod photoreceptors.^22^ Notwithstanding, there were also significant improvements in red light sensitivity. The changes in the FST are highly relevant, as the phase 3 trial showed a strong correlation between improvements in the FST and changes in the bilateral multi-luminance mobility test,^21^ which attempts to provide a clinically meaningful and reliable measure of functional vision in patients with low vision and nyctalopia.^23^ Because the multi-luminance mobility test was not adopted in clinical practice, our findings support adoption of the FST test to evaluate efficacy.

Remarkable improvement was seen in the visual field in all four eyes. Compared with baseline, in which there were reduced or no responses only to Goldman III4e or I4e stimuli, the visual field was notably expanded post-injection in all four eyes with responses to Goldman visual fields V4e, III4e, and I4e. Although there was some variance at the different follow-up times, the changes in visual field appeared to be largely stable. Benefits in visual field were also documented by previous phase 1^24^ and phase 3^5^ trials. In the former, it was noted that Goldman V4e data showed high inter-visit variability,^24^^,^^25^ whereas Goldman III4e showed large gains in the phase 3 results.^5^ The combined results of Goldman testing thus indicate that there is a much larger area of retinal sensitivity, which can be attributed to amelioration in photoreceptor function, corresponding to benefits in peripheral vision that likely correlate with better navigational ability.

All eyes with unstable fixation also showed improvement in fixation stability, evaluable by reduction of BCEA area using microperimetry. In clinical trials, microperimetry has been adopted to assess changes in retinal sensitivity and may reflect differences in nystagmus.^26^^,^^27^ Our findings suggest that BCEA evaluation can highlight changes in fixation stability after treatment, which may correlate to improvements in nystagmus. Actually, improvements in nystagmus were reported in a phase 1 trial^22^ and were stable during long-term follow-up.^25^

On basis of our previous investigations on white light stimulus pupillometry,^25^^,^^28^^,^^29^ we evaluated outcomes of VN treatment with chromatic pupillometry. Although white light stimulus pupillometry detects the summation response of all light-sensitive cells within the retina by measuring the resulting transient pupil constriction, chromatic pupillometry is increasingly used to evaluate retinal disorders^30^^–^^34^ and has also been used to objectively measure the function of rods and cones in patients with mutations in *RPE65*.^18^ Both chromatic and white stimulus pupillometry showed increases in maximum pupillary constriction that ranged from 10% to 20% depending on the stimulus. In particular, improvement was seen for blue and white light across all intensities and for red light at lower intensities. Chromatic pupillometry thus provides additional information compared with white stimulus pupillometry and reinforces that VN therapy is also associated with improved functioning of cone cells as suggested from a mouse model.^35^

VN treatments of the first two Italian patients in clinical practice showed significant improvement in visual function in all treated eyes in terms of visual acuity (i.e., at least one ETDRS lines), visual field (i.e., area enlargement of at least 20%), light sensitivity threshold (i.e., at least 10 dB). Of note, surgical complications in the treatment of the second patient did not prevent significant improvements, even if the changes were observed at later follow-up time points. In particular, despite the subretinal hemorrhage in the first treated eye, patient 2 showed a stable BCVA and significant benefit of FST at day 15, which was corroborated by improvements observed with other investigations at the following time points (e.g., SKVF performed at day 45, after resolution of subretinal hemorrhage). These findings are in line with the reported cases of spontaneous absorption of small-size subretinal hemorrhage with recovery of visual function.^36^^–^^38^

To the best of our knowledge, this is the first study to use chromatic pupillometry to investigate changes in visual function in patients undergoing gene therapy for *RPE65*-IRDs and thus provides further insight into the retinal changes that occur following treatment. Importantly, the benefits in visual function were stable over the 6-month observation period, in agreement with previous studies reporting stability for up to 4 years.^21^^,^^25^^,^^39^

In conclusion, although follow-up for longer periods of time is needed to better understand the effectiveness of therapy over the very long term, the outcomes observed in the first Italian patients undergoing this therapy nonetheless confirm the results of clinical trials, with no safety issues and clinical improvements in visual function.
